# Supplementary material for: Naphthoquinone-derivative as a synthetic compound to overcome the antibiotic resistance of methicillin-resistant S. aureus
Source: Commun Biol. 2020 Sep 24;3:529. doi: 10.1038/s42003-020-01261-0 (PMC7518446; doi:10.1038/s42003-020-01261-0)
Supplement: Supplementary file 1 — Supplementary Information [file 42003_2020_1261_MOESM1_ESM.pdf]

Supplementary Information

Naphthoquinone-derivative as a synthetic compound to overcome the antibiotic resistance of methicillin-resistant *S. aureus*

Ronghui Song[1], Bing Yu[2], Dirk Friedrich[1], Junfeng Li[1], Hao Shen[1], Harald Krautscheid[3], Songping D. Huang[1]*, and Min-Ho Kim[2]*

Supplementary Methods

Synthesis and characterization of lawsone derivatives

Materials and measurement method

The chemical reagents used in this study were purchased from Sigma-Aldrich, Acros Organic, Alfa Aesar, TCI America, and Fisher Scientific without further purification. Lawsone, vancomycin and ciprofloxacin were purchased from Sigma-Aldrich. Daptomycin and ofloxacin were purchased from TCI America. Deuterium solvents were bought from Cambridge isotope. The nuclear magnetic resonance spectrums were acquired from a Bruker DMX 400 MHz NMR (Frequency: 400 MHz for ^{1}H-NMR; 100 MHz for ^{13}C-NMR). High Resolution mass spectra were obtained using an Exactive Plus mass spectrometer (Thermo Scientific, Bremen, Germany). The lawsone derivatives were measured as pure solids ionized by electronically excited Helium gas using an ID-cube DART-source (Ionsense, Saugus, MA, USA). Isotopic patterns were simulated using the software Thermo Xcalibur 3.0.63 (Thermo Scientific, Bremen, Germany). X-ray diffraction data were obtained using two different diffractometer systems (STOE, Darmstadt, Germany). The first system used is a IPDS-2T image plate diffractometer using Mo-K_{α} radiation (λ = 71.073 pm). The second system used is a STADIVARI diffractometer equipped with reverse-biased silicon diode array detector using Cu-K_{α} radiation (λ = 154.186 pm). The data sets were processed with the program STOE X-AREA.[1] The structures were solved by direct methods[2] and refined using SHELX-2018.[3] The non-hydrogen atoms of the frameworks were refined anisotropically. The coordinates of the hydrogen atoms of the frameworks were calculated for idealized positions. The programs DIAMOND 3.2f and Mercury[4] were used for visualization the structures.

General procedure for synthesizing **3a** and **3b**[5-6]:

Copper(I) iodide (0.042 g, 0.220 mol) was added to a mixture containing 4-bromobenzaldehyde (1.85 g, 10.0 mmol), bis(triphenylphosphine)palladium (II) dichloride (0.077 g, 0.110 mmol) and 1-hexyne (0.986 g, 12.0 mmol) or 1-octyne (1.32 g, 12.0 mmol) in Et_3N (20 mL). The mixture was vigorously stirred overnight at 50 °C under N_2 and then the reaction mixture was cooled to room temperature and filtered through Celite to remove insoluble materials. The filtrate was washed with brine (3×20 mL) and dried over anhydrous $MgSO_4$. The solvent was removed by rotary evaporator and the resulting products was purified by flash column chromatography (silica gel, hexane as eluent) to afford final products. The final products were not stable under air, so further purification was not performed, and the products were directly used in the next reaction.

4-(1-Hexyn-1-yl)benzaldehyde (**3a**): Yellow oil; Yield 1.62 g (87 %).1H NMR (400 MHz, CDCl3):δ(ppm) = 0.90 (t, *J*=7.2 Hz, 3H, $–CH_3$), 1.43-1.67 (m, 4H, $–CH_2–$), 2.44 (t, *J*=6.9 Hz, 2H,–C≡C–CH_2–), 7.51 (d, *J*=8.3 Hz, 2H, Ar-H), 7.78 (d, J=8.3 Hz, 2H, Ar-H), 9.97 (s, 1H, –CHO).

4-(1-Octyn-1-yl)benzaldehyde (**3b)**: Yellow oil; Yield 1.79 g (83 %) ^{1}H NMR (400 MHz, $CDCl_3$) δ(ppm) = 0.90 (t, *J*=7.1 Hz, 3H, $–CH_3$), 1.28–1.64 (m, 8H, $–CH_2–$), 2.44 (t, *J*=7.1 Hz, 2H,–C≡C–CH_2–), 7.53 (d, *J*=8.3 Hz, 2H, Ar-H), 7.80 (d, *J*=8.3 Hz, 2H, Ar-H), 9.99 (s, 1H, –CHO).

General procedure for synthesizing **4d** and **4e**[7]**:**

The **3a** (1.62 g, 8.70 mmol) which was dissolved in ethyl acetate (20 mL) was introduced with H_2 gas by a syringe needle and after 5 minutes, palladium/activated carbon (Pd 10%) (0.080 g) was added into this solution. The mixture was stirred for 6 h under H_2, and then, allowed to pass through a filter with Celite on it and washed with hexane (2×100 mL) thoroughly. The solvent of filtrate

was removed by rotary evaporator and the resulting dark yellow oil was purified by silica gel flash column chromatography (eluent; hexane/ethyl acetate, 20:1, v/v) to afford final product (**4d**). The product was used in the next reaction without further purification due to instability under air. **4e** was synthesized use the similar procedure above.

4-Hexylbenzaldehyde (**4d**): Yellow oil; Yield 1.36 g (82 %).^{1}H NMR (400 MHz, $CDCl_3$) δ(ppm) = 0.87 (t, *J*=7.5 Hz, 3H, $-CH_3$), 1.31 (br, 6H), 1.63–1.57 (m, 2H), 2.68 (t, *J*=7.5, 2H, Ar-CH_2), 7.34 (d, *J*=7.5 Hz, 2H) 7.80 (d, *J*=7.5 Hz, 2H), 9.97 (s, 1H, –CHO). ^{1}H-NMR was in agreement with those reported [8].

4-Octylbenzaldehyde (**4e**): Yellow oil; Yield 1.39 g (76 %). ^{1}H NMR (400 MHz, $CDCl_3$) δ(ppm) = 0.88 (t, *J*=7.1 Hz, 3H, $–CH_3$), 1.26–1.32 (m, 10H, $–CH_2–$), 1.61–1.67 (m, 2H, $–CH_2–$), 2.69 (t, *J*=7.1 Hz, 2H, Ar-CH2–), 7.34 (d, *J*=7.8 Hz, 2H, Ar-H), 7.80 (d, *J*=7.8 Hz, 2H, Ar-H), 9.97 (s, 1H,–CHO).

<u>General procedure for synthesizing **6a-6e**[9]</u>**:**

L-proline (1.0 mmol) was added to 50 mL dichloromethane containing corresponding aldehyde (10 mmol), 2-hydroxy-1,4- naphthoquinone (5.0 mmol) and Hantzsch ester (6.0 mmol). This mixture was vigorously stirred at room temperature for 24 h and the resulting solution was passed through a filter with Celite mounted. The Celite was thoroughly washed with ethyl acetate (3×200 mL) and the filtrate was concentrated by using rotary evaporator. The final pure product was obtained by flash column chromatography (silica gel, hexane and ethyl acetate as eluents).

2-Benzyl-3-hydroxy-[1,4]naphthoquinone (**6a**): Yellow solid; Yield: 74%; ^{1}H NMR (400 MHz, $CDCl_3$) δ(ppm) = 8.13 (1H, d, *J* = 7.5 Hz), 8.07 (1H, d, *J* = 7.5 Hz), 7.75 (1H, dt, *J* = 7.5, 1.0 Hz), 7.68 (1H, dt, *J* = 7.5, 1.0 Hz), 7.42 (2H, d, *J* = 7.0 Hz), 7.28 (2H, t, *J* = 7.5 Hz), 7.20 (1H, t, *J* = 7.5 Hz), 3.97 (2H, s); ^{13}C NMR ($CDCl_3$, 100 MHz) δ 184.4 (C, C=O), 181.7 (C, C=O), 153.0 (C), 138.9 (C), 135.0 (CH), 133.0 (CH), 132.8 (C), 129.4 (C), 129.2 (2 x CH), 128.4 (2 x CH), 126.9 (CH), 126.3 (CH), 126.1 (CH), 123.0 (C), 29.1 (CH_2). ESI-HRMS m/z: $[M+H]^+$; Calcd for $[C_{17}H_{12}O_3+H]^+$ 265.0859; found 265.0857. (Supplementary Figure 1)

2-[(4-Ethylphenyl)methyl]-3-hydroxy-[1,4]naphthoquinone (**6b**): Yellow solid; Yield: 66%; ^{1}H NMR (400 MHz, $CDCl_3$) δ(ppm) = 8.11 (dd, *J* = 7.7, 1.3 Hz, 1H), 8.06 (dd, *J* = 7.6, 1.1 Hz, 1H), 7.74 (td, *J* = 7.6, 1.4 Hz, 1H), 7.66 (td, *J* = 7.5, 1.3 Hz, 1H), 7.40 (s, 1H), 7.31 (d, *J* = 8.1 Hz, 2H), 7.09 (d, *J* = 8.2 Hz, 2H), 3.91 (s, 2H), 2.58 (q, *J* = 7.6 Hz, 2H), 1.19 (t, *J* = 7.6 Hz, 3H); ^{13}C NMR ($CDCl_3$, 100 MHz) δ 184.4 (C, C=O), 181.7 (C, C=O), 152.9 (C), 142.3 (C), 136.1 (CH), 135.0 (CH), 132.9 (C), 129.4 (C), 129.1 (2 x CH), 127.9 (2 x CH), 126.9 (CH), 126.1 (CH), 123.3 (C), 28.7 (CH_2), 28.4 (CH_2), 15.5 (CH_3). ESI-HRMS m/z: $[M+H]^+$; Calcd for $[C_{19}H_{16}O_3+H]^+$ 293.1172; found 293.1169. (Supplementary Figure 2)

2-[(4-butylphenyl)methyl]-3-hydroxy-[1,4]naphthoquinone (**6c**): Yellow solid; Yield: 32%; ^{1}H NMR (400 MHz, $CDCl_3$) δ(ppm) = 8.12 (dd, *J* = 7.7, 0.9 Hz, 1H), 8.06 (dd, *J* = 7.6, 1.0 Hz, 1H), 7.74 (td, *J* = 7.6, 1.4 Hz, 1H), 7.67 (td, *J* = 7.5, 1.4 Hz, 1H), 7.39 (s, 1H), 7.30 (d, *J* = 8.1 Hz, 1H), 7.07 (d, *J* = 8.1 Hz, 2H), 3.91 (s, 2H), 2.59 – 2.50 (m, 2H), 1.62 – 1.45 (m, 2H), 1.32 (dq, *J* = 14.6, 7.3 Hz, 2H), 0.89 (t, *J* = 7.3 Hz, 2H).; ^{13}C NMR ($CDCl_3$, 100 MHz) δ 184.3 (C, C=O), 181.6 (C, C=O), 152.9 (C), 140.9 (C), 136.0 (CH), 135.0 (CH), 132.9 (C), 132.8 (C), 129.4 (2 x CH), 129.0

(2 x CH), 128.5 (CH), 126.9 (CH), 126.1 (CH), 123.3 (C), 35.2 (CH_2), 33.6 (CH_2), 28.7 (CH_2), 22.4 (CH_2), 13.9 (CH_3). ESI-HRMS m/z: $[M+H]^+$; Calcd for $[C_{21}H_{20}O_3+H]^+$ 321.1485; found 321.1482. (Supplementary Figure 3)

2-[(4-hexylphenyl)methyl]-3-hydroxy-[1,4]naphthoquinone (**6d**): Yellow solid; Yield: 35%; ^{1}H NMR (400 MHz, $CDCl_3$) δ(ppm) = 8.11 (dd, *J* = 7.7, 0.9 Hz, 1H), 8.06 (dd, *J* = 7.6, 1.0 Hz, 1H), 7.74 (td, *J* = 7.6, 1.4 Hz, 1H), 7.66 (td, *J* = 7.5, 1.4 Hz, 1H), 7.39 (s, 1H), 7.29 (d, *J* = 8.1 Hz, 2H), 7.07 (d, *J* = 8.1 Hz, 2H), 3.91 (s, 3H), 2.68 – 2.37 (m, 3H), 1.55 (p, *J* = 7.6 Hz, 2H), 1.34-1.22 (m, 6H), 0.86 (t, *J* = 6.5 Hz, 3H); ^{13}C NMR ($CDCl_3$, 100 MHz) δ 184.4 (C, C=O), 181.7 (C, C=O), 152.9 (C), 141.0 (C), 136.0 (CH), 135.0 (CH), 132.9 (C), 129.4 (C), 129.0 (2 x CH), 128.5 (2 x CH), 126.9 (CH), 126.1 (CH), 123.3 (C), 29.1 (CH_2), 35.6 (CH_2), 31.7 (CH_2), 31.5 (CH_2), 29.1 (CH_2), 28.7 (CH_2), 22.6 (CH_2), 14.1 (CH_3). ESI-HRMS m/z: $[M+H]^+$; Calcd for $[C_{23}H_{24}O_3+H]^+$ 349.1798; found 349.1796. (Supplementary Figure 4)

2-[(4-octylphenyl)methyl]-3-hydroxy-[1,4]naphthoquinone (**6e**): Yellow solid; Yield: 35%; ^{1}H NMR (400 MHz, $CDCl_3$) δ(ppm) = 8.11 (ddd, *J* = 7.7, 1.4, 0.5 Hz, 1H), 8.05 (ddd, *J* = 7.6, 1.4, 0.5 Hz, 1H), 7.73 (td, *J* = 7.6, 1.4 Hz, 1H), 7.65 (td, *J* = 7.5, 1.4 Hz, 1H), 7.41 (s, 1H), 7.29 (d, *J* = 8.2 Hz, 1H), 7.06 (d, *J* = 8.3 Hz, 1H), 3.91 (s, 2H), 2.69 – 2.32 (m, 2H), 1.55 (p, *J* = 7.6 Hz, 2H), 1.36 – 1.20 (m, 10H), 0.85 (t, *J* = 6.5 Hz, 2H).; ^{13}C NMR ($CDCl_3$, 100 MHz) δ 184.4 (C, C=O), 181.7 (C, C=O), 153.0 (C), 141.0 (C), 136.0 (CH), 134.9 (CH), 132.9 (C), 129.4 (C), 129.0 (2 x CH), 128.5 (2 x CH), 126.9 (CH), 126.1 (CH), 123.3 (C), 35.6 (CH_2), 31.9 (CH_2), 31.5 (CH_2), 29.5 (CH_2), 29.4 (CH_2), 29.2 (CH_2), 28.7 (CH_2), 22.7 (CH_2), 14.1 (CH_3). ESI-HRMS m/z: $[M+H]^+$; Calcd for $[C_{25}H_{28}O_3+H]^+$ 377.2111; found 377.2110. (Supplementary Figure 5)

Synthesis of iron-6c complex.

The iron-**6c** complex was synthesized as reported previously with modifications.[10] An aqueous solution (10 mL) containing iron (III) nitrate nonahydrate (3 mmol) was added dropwise to a solution (MeOH/H_2O, 1/1, v/v, 50 mL) containing **6c** (1 mmol). After addition, 1 N NaOH solution was used to adjust the pH of the solution to 7.4, and this solution was vigorously stirred for 2 hours. The red-black precipitation was filtered, and the product was washed several times by using MeOH and deionized water. The final product was recrystallized in EtOAc/DCM with a yield of 27%.

The molecular structure of this complex was validated by X-ray single crystal structure analysis. The iron, oxygen and carbon atoms were found, and the connectivity of the atoms was verified. A visualization of the iron-**6c** complex was generated from the crystallographic information (Supplementary Figure 7a). The identity of iron-**6c** complex was confirmed by MALDI-MS, MALDI-MS m/z: $[M+Ag]^+$; Calcd for $[C_{42}H_{38}FeO_6+Ag]^+$ 801.244; found 801.107 (Supplementary Figure 7b).

The atom coordinates and displacement parameters of the iron-**6c** complex were extracted from res-file and given in Supplementary Data 1.

Supplementary Figures

a

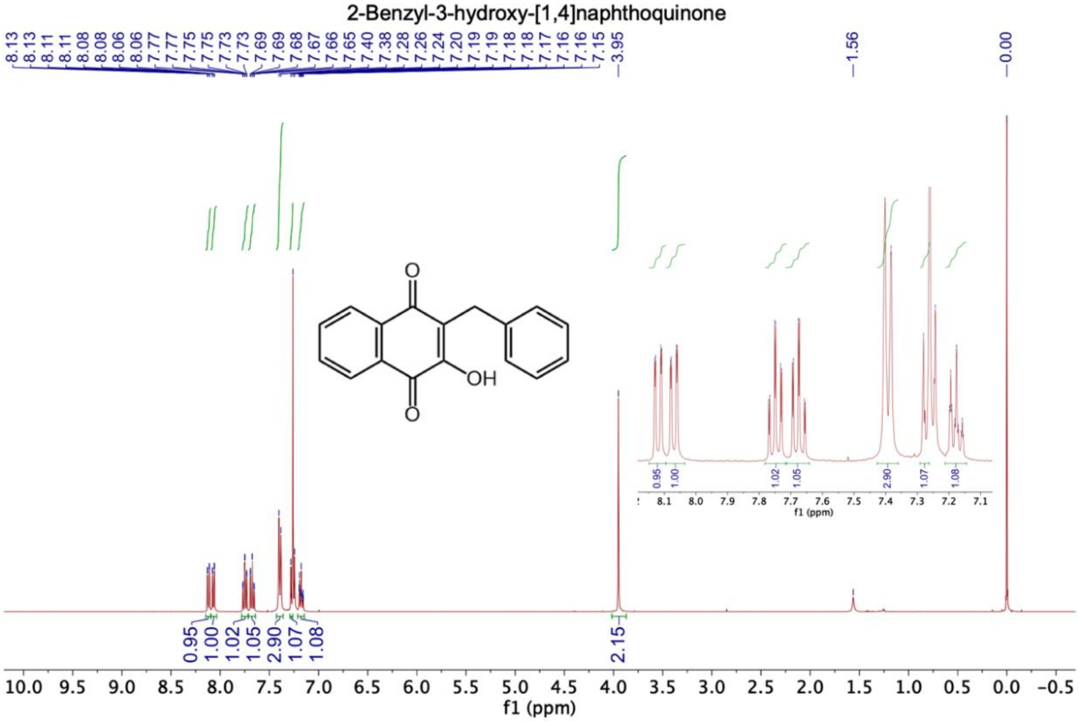

b

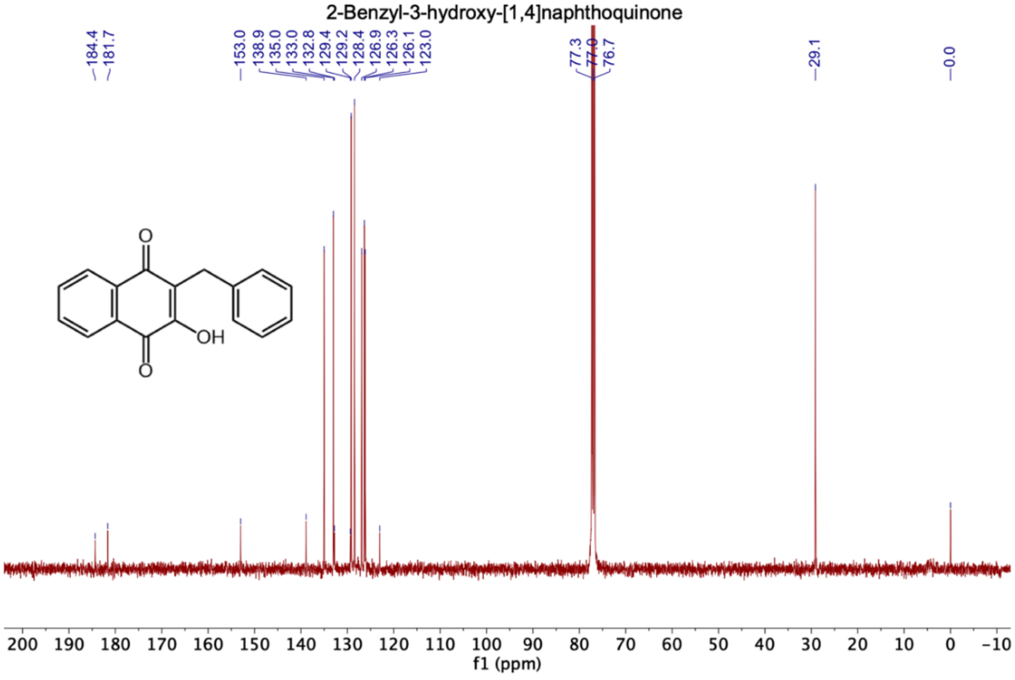

c

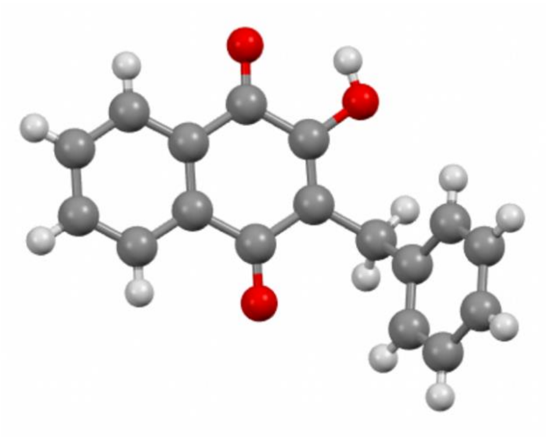

Empirical formula	$C_{17} H_{12} O_3$
T(K)	180
Space group	*P* 2_1/n (14)
a/Å	6.0377(2)
b/Å	9.5318(4)
c/Å	22.0493(7)
Cell angles	α 90 β 94.968(3) γ 90
Cell volume (Å^3)	1264.17
Z	4
R-Factor (%)	3.04
Density	1.388

d

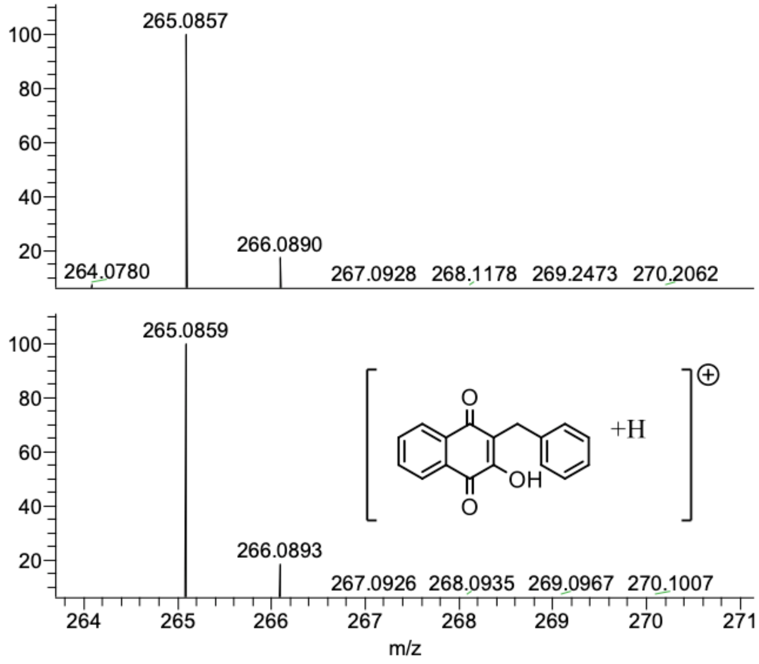

Supplementary Figure 1. Characterization of compound 6a. (a) ^{1}H NMR spectrum of compound **6a** in $CDCl_3$. **(b)** ^{13}C NMR spectrum of compound **6a** in $CDCl_3$. **(c)** Molecular structure of **6a** (left) and its crystal data (right). CCDC deposition number: 1989181. **(d)** HRMS Spectrum. Top: signal as measured. Bottom: simulation of $[C_{17}H_{12}O_3+H]^+$.

a

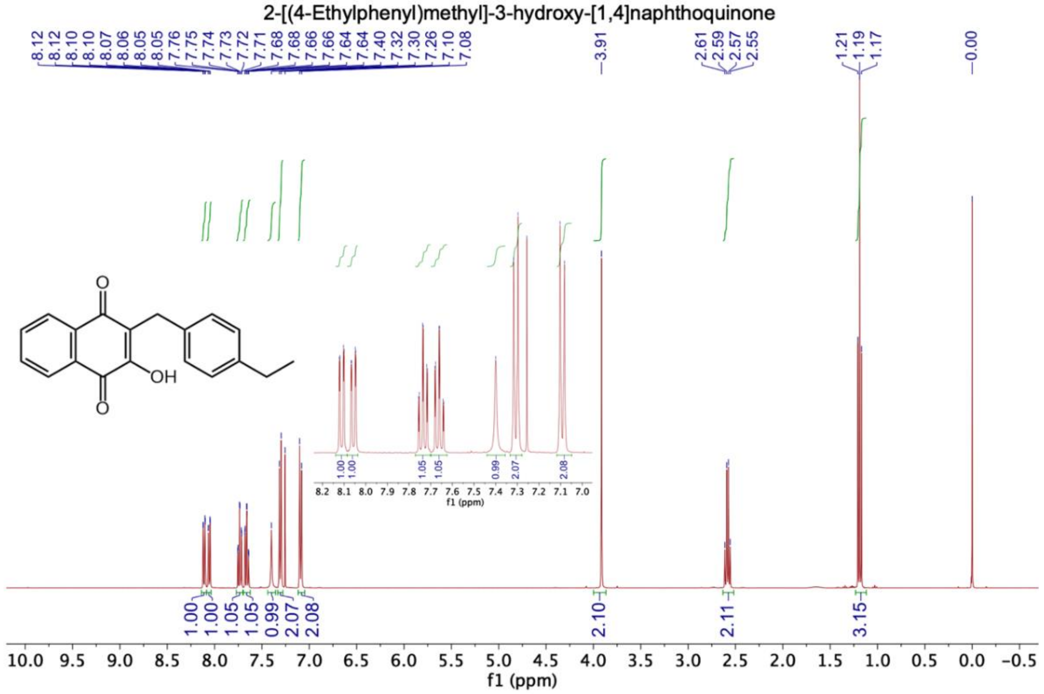

2-[(4-Ethylphenyl)methyl]-3-hydroxy-[1,4]naphthoquinone
8.12
8.12
8.10
8.07
8.06
8.05
7.76
7.75
7.74
7.73
7.72
7.71
7.68
7.68
7.66
7.66
7.64
7.64
7.40
7.32
7.30
7.26
7.10
7.08
3.91
2.61
2.59
2.57
2.55
1.21
1.19
1.17
-0.00
OH
1.00
1.00
1.05
1.05
0.99
2.07
2.08
2.10
2.11
3.15
f1 (ppm)

b

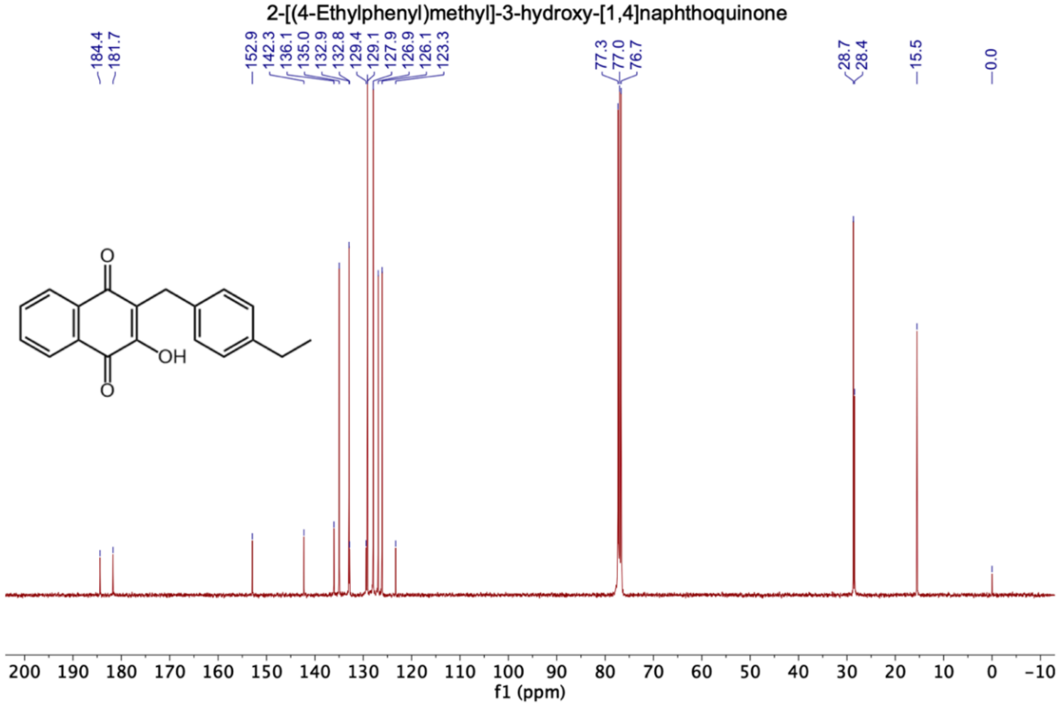

2-[(4-Ethylphenyl)methyl]-3-hydroxy-[1,4]naphthoquinone
184.4
181.7
152.9
142.3
136.1
135.0
132.9
132.8
129.4
129.1
127.9
126.9
126.1
123.3
77.3
77.0
76.7
28.7
28.4
15.5
-0.0
OH
f1 (ppm)

c

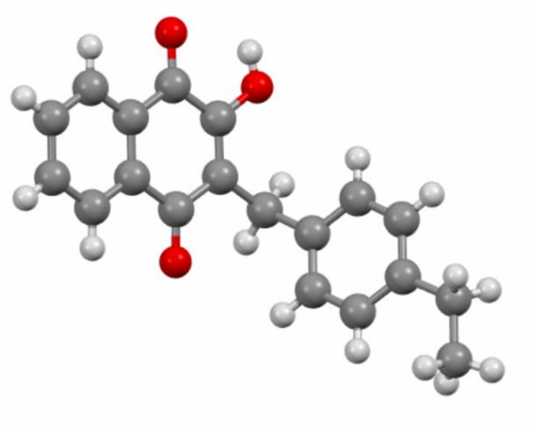

Empirical formula	$C_{19}H_{16}O_3$
T(K)	180
Space group	*P* 2_1/n (14)
a/Å	25.2151(6)
b/Å	5.8017(2)
c/Å	30.3926(8)
Cell angles	α 90 β 100.218(2) γ 90
Cell volume (Å³)	4375.63
Z	12
R-Factor (%)	4.42
Density	1.331

d

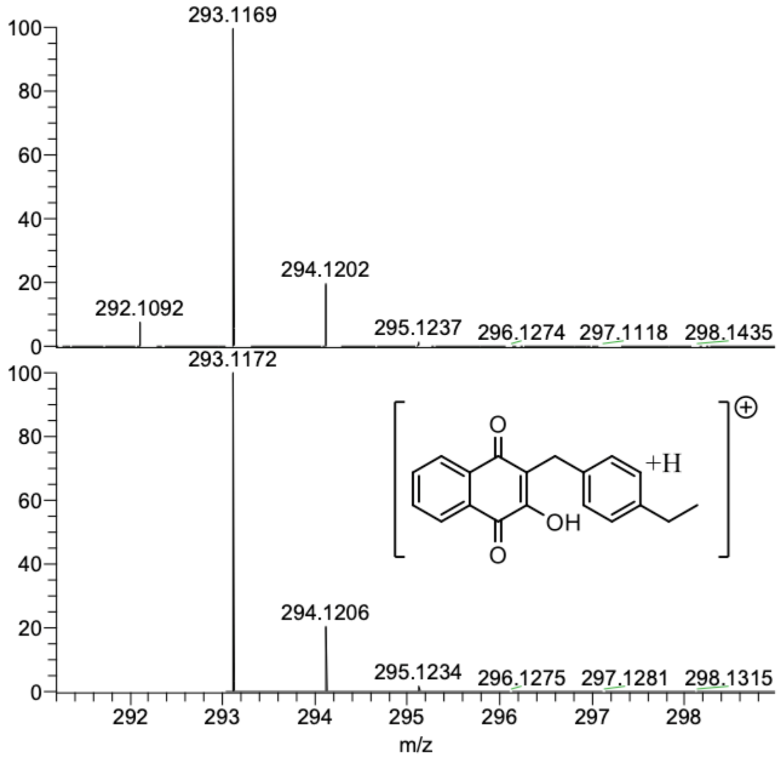

Supplementary Figure 2. Characterization of compound 6b. **(a)** ^{1}H NMR spectrum of compound **6b** in $CDCl_3$. **(b)** ^{13}C NMR spectrum of compound **6b** in $CDCl_3$. **(c)** Molecular structure of **6b** (left) and its crystal data (right). CCDC deposition number: 1989182. **(d)** HRMS Spectrum. Top: signal as measured. Bottom: simulation of $[C_{19}H_{16}O_3+H]^+$.

a

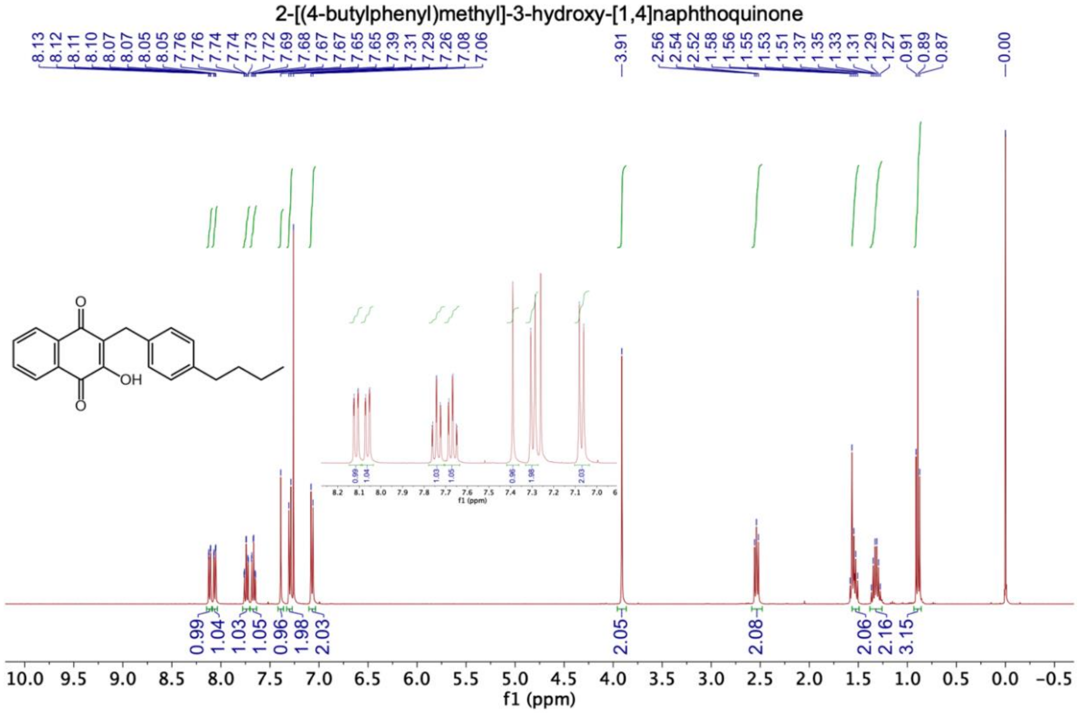
2-[(4-butylphenyl)methyl]-3-hydroxy-[1,4]naphthoquinone
8.13 8.12 8.11 8.10 8.07 8.05 8.05 7.76 7.76 7.74 7.74 7.73 7.72 7.69 7.68 7.67 7.67 7.65 7.65 7.39 7.31 7.29 7.26 7.08 7.06
3.91
2.56 2.54 2.52 1.58 1.56 1.55 1.53 1.51 1.37 1.35 1.33 1.31 1.29 1.27 0.91 0.89 0.87
0.00
OH
0.99 1.04 1.03 1.05 0.96 1.98 2.03 2.05 2.08 2.06 2.16 3.15
10.0 9.5 9.0 8.5 8.0 7.5 7.0 6.5 6.0 5.5 5.0 4.5 4.0 3.5 3.0 2.5 2.0 1.5 1.0 0.5 0.0 −0.5
f1 (ppm)

b

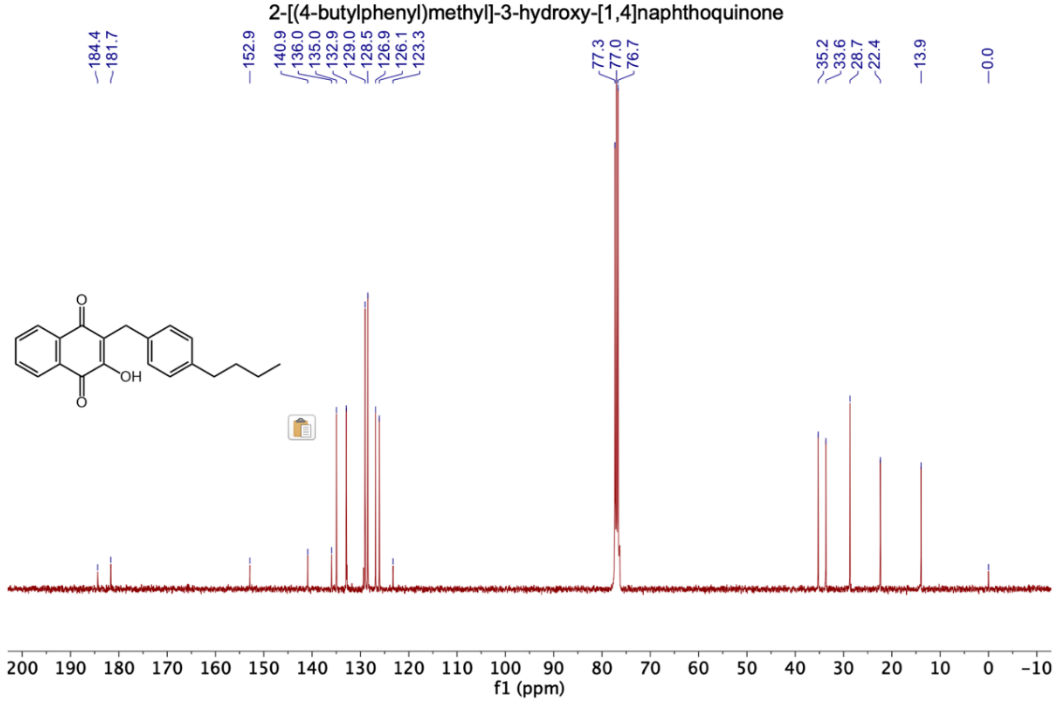
2-[(4-butylphenyl)methyl]-3-hydroxy-[1,4]naphthoquinone
184.4 181.7
152.9
140.9 136.0 135.0 132.9 129.0 128.5 126.9 126.1 123.3
77.3 77.0 76.7
35.2 33.6 28.7 22.4
13.9
0.0
OH
200 190 180 170 160 150 140 130 120 110 100 90 80 70 60 50 40 30 20 10 0 −10
f1 (ppm)

c

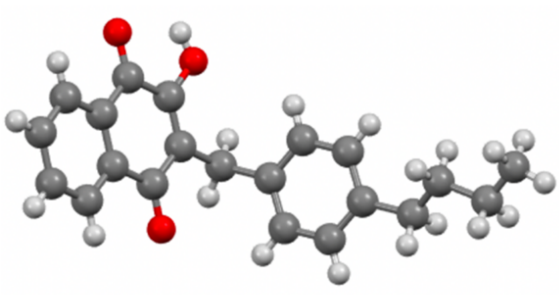

Empirical formula	$C_{21} H_{20} O_3$
T(K)	200
Space group	*P* 1 (2)
a/Å	6.0316(8)
b/Å	9.9372(13)
c/Å	14.865(2)
Cell angles	α 105.273(10)<br>β 97.989(11)<br>γ 92.865(10)
Cell volume (Å³)	847.654
Z	2
R-Factor (%)	4.81
Density	1.255

d

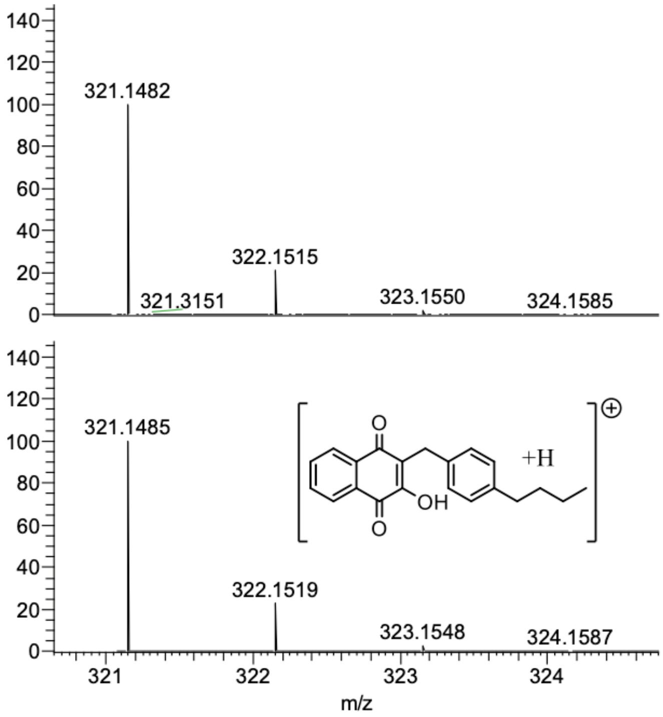

Supplementary Figure 3. Characterization of compound 6c. (a) ^{1}H NMR spectrum of compound **6c** in $CDCl_3$. **(b)** ^{13}C NMR spectrum of compound **6c** in $CDCl_3$. **(c)** Molecular structure of **6c** (left) and its crystal data (right). CCDC deposition number: 1989180. **(d)** HRMS Spectrum. Top: signal as measured. Bottom: simulation of $[C_{21}H_{20}O_3+H]^+$.

a

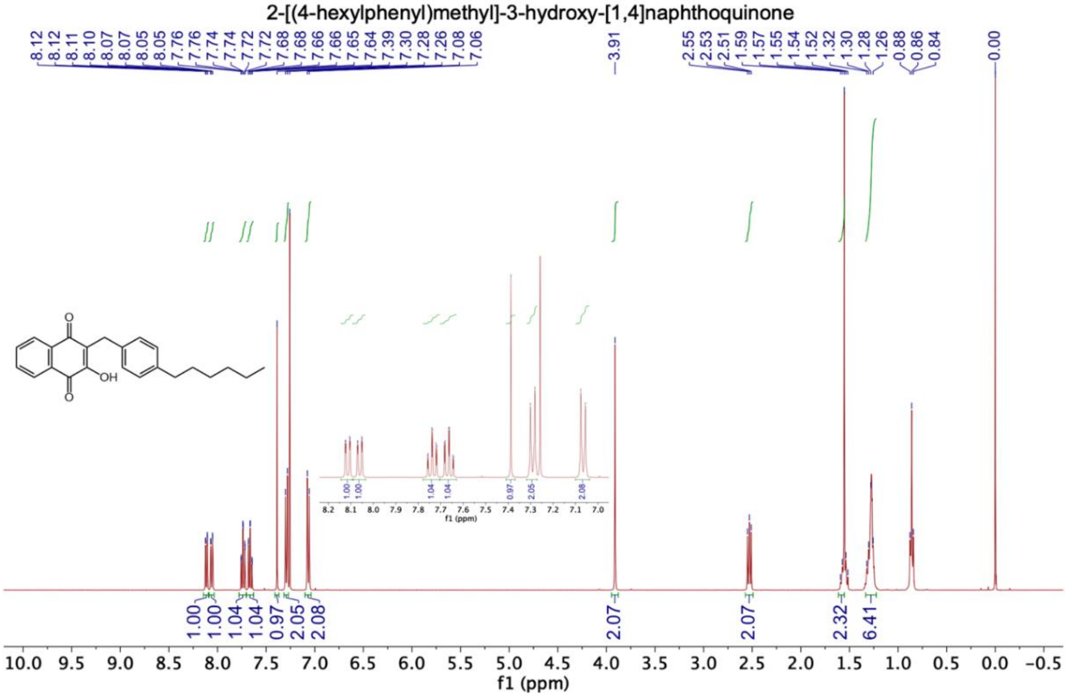
2-[(4-hexylphenyl)methyl]-3-hydroxy-[1,4]naphthoquinone
8.12 8.12 8.11 8.10 8.07 8.07 8.05 8.05 7.76 7.76 7.74 7.74 7.72 7.72 7.68 7.68 7.66 7.66 7.65 7.64 7.39 7.30 7.28 7.26 7.08 7.06
3.91
2.55 2.53 2.51 1.59 1.57 1.55 1.54 1.52 1.32 1.30 1.28 1.26 0.88 0.86 0.84
0.00
OH
1.00 1.00 1.04 1.04 0.97 2.05 2.08
2.07
2.07
2.32 6.41
f1 (ppm)

b

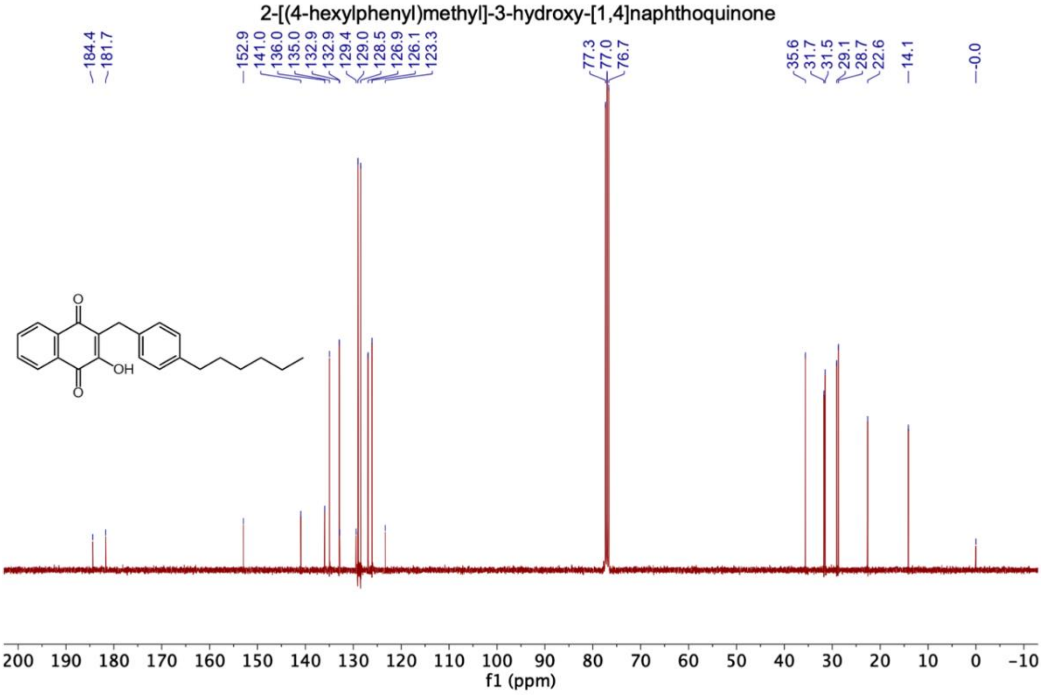
2-[(4-hexylphenyl)methyl]-3-hydroxy-[1,4]naphthoquinone
184.4 181.7
152.9 141.0 136.0 135.0 132.9 132.9 129.4 129.0 128.5 126.9 126.1 123.3
77.3 77.0 76.7
35.6 31.7 31.5 29.1 28.7 22.6
14.1
0.0
OH
f1 (ppm)

c

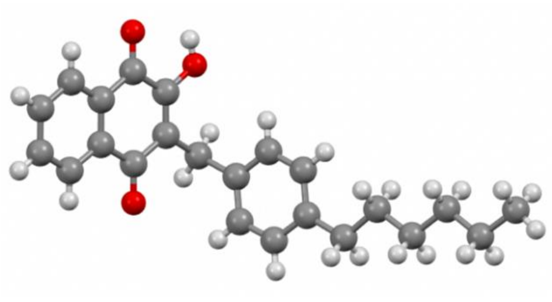

Empirical formula	$C_{23} H_{24} O_3$
T(K)	180
Space group	*P* 1 (2)
a/Å	6.1849(3)
b/Å	9.2401(5)
c/Å	16.8019(9)
Cell angles	α 104.184(4)<br>β 89.792(4)<br>γ 91.379(4)
Cell volume (Å³)	930.668
Z	2
R-Factor (%)	3.8
Density	1.243

d

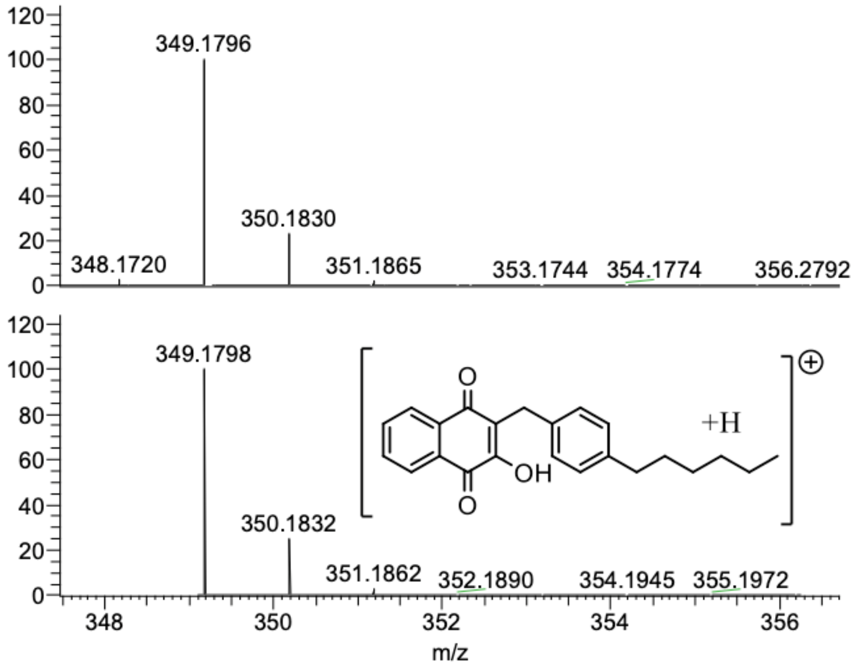

Supplementary Figure 4. Characterization of compound 6d. **(a)** ^{1}H NMR spectrum of compound **6d** in $CDCl_3$. **(b)** ^{13}C NMR spectrum of compound **6d** in $CDCl_3$. **(c)** Molecular structure of **6d** (left) and its crystal data (right). CCDC deposition number: 1989183. **(d)** HRMS Spectrum. Top: signal as measured. Bottom: simulation of $[C_{23}H_{24}O_3+H]^+$.

a

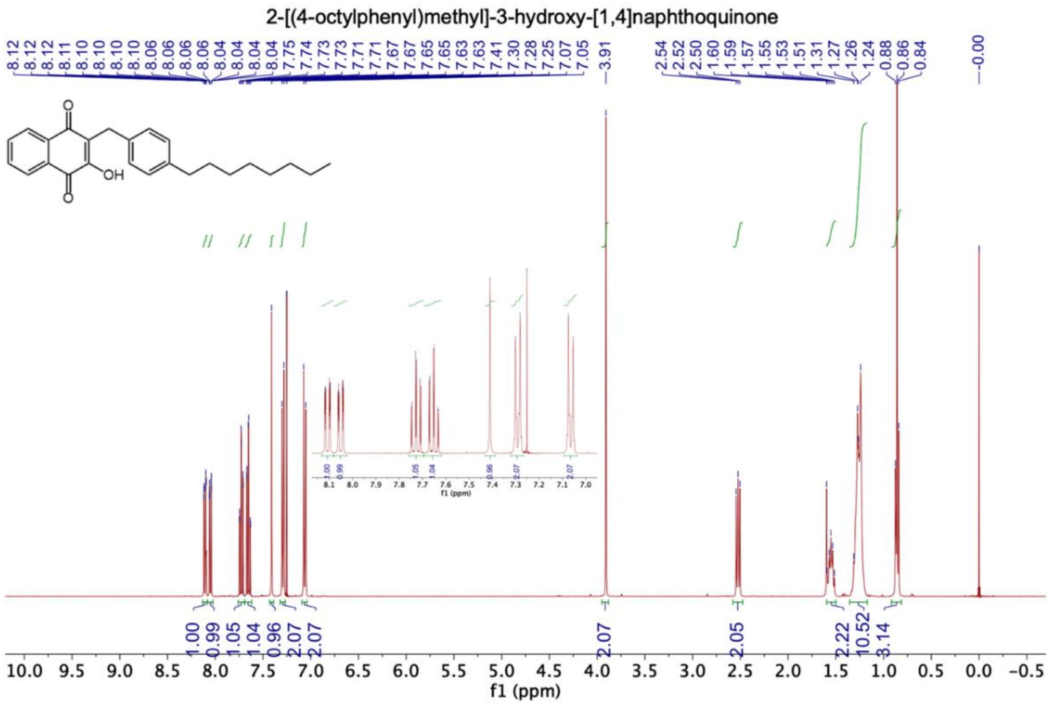

2-[(4-octylphenyl)methyl]-3-hydroxy-[1,4]naphthoquinone
8.12 8.12 8.12 8.11 8.10 8.10 8.10 8.10 8.06 8.06 8.06 8.06 8.04 8.04 8.04 8.04 7.75 7.74 7.73 7.73 7.71 7.71 7.67 7.67 7.65 7.65 7.63 7.63 7.41 7.30 7.28 7.25 7.07 7.05
3.91
2.54 2.52 2.50 1.60 1.59 1.57 1.55 1.53 1.51 1.31 1.27 1.26 1.24 0.88 0.86 0.84
-0.00
OH
1.00 0.99 1.05 1.04 0.96 2.07 2.07 2.07 2.05 2.22 10.52 3.14
f1 (ppm)

b

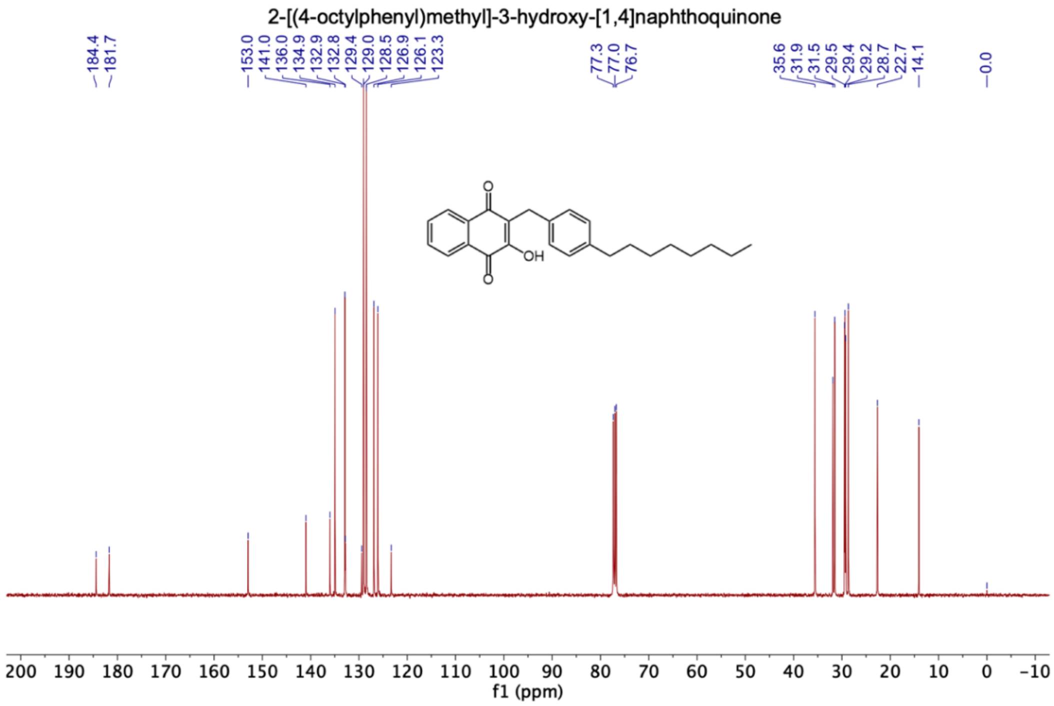

2-[(4-octylphenyl)methyl]-3-hydroxy-[1,4]naphthoquinone
184.4 181.7
153.0 141.0 136.0 134.9 132.9 132.8 129.4 129.0 128.5 126.9 126.1 123.3
77.3 77.0 76.7
35.6 31.9 31.5 29.5 29.4 29.2 28.7 22.7 14.1
0.0
OH
f1 (ppm)

c

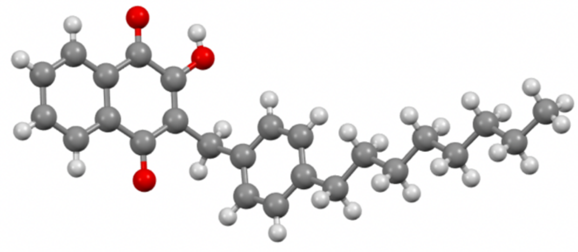

Empirical formula	C_{25} H_{28} O_3
T(K)	180
Space group	*P* 1 (2)
a/Å	6.1641(4)
b/Å	9.1683(5)
c/Å	18.5147(15)
Cell angles	α 98.977(6)<br>β 95.041(6)<br>γ 90.776(6)
Cell volume (Å^3)	1029.14
Z	2
R-Factor (%)	6.2
Density	1.215

d

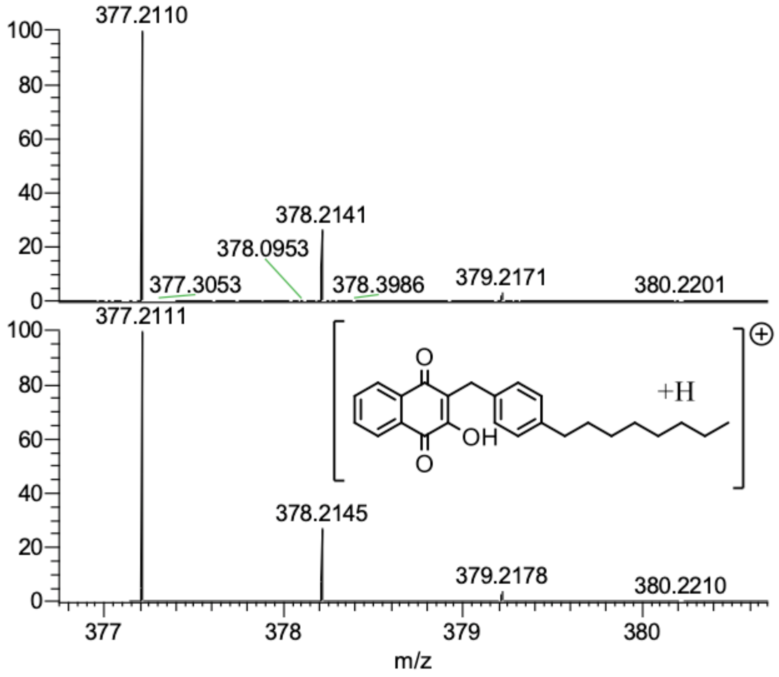

Supplementary Figure 5. Characterization of compound 6e. (a) ^{1}H NMR spectrum of compound **6e** in $CDCl_3$. **(b)** ^{13}C NMR spectrum of compound **6e** in $CDCl_3$. **(c)** Molecular structure of **6e** (left) and its crystal data (right). CCDC deposition number: 1989184. **(d)** HRMS Spectrum. Top: signal as measured. Bottom: simulation of $[C_{25}H_{28}O_3+H]^+$.

a

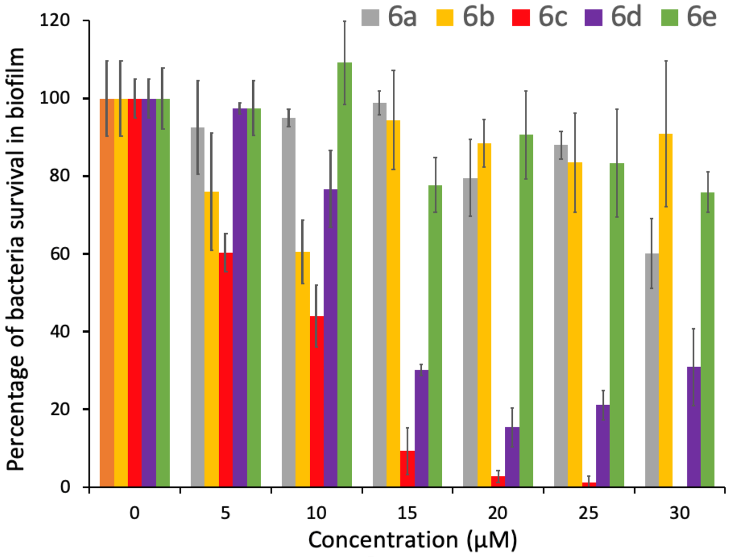
6a 6b 6c 6d 6e
Percentage of bacteria survival in biofilm
Concentration (μM)
0 20 40 60 80 100 120
0 5 10 15 20 25 30

b

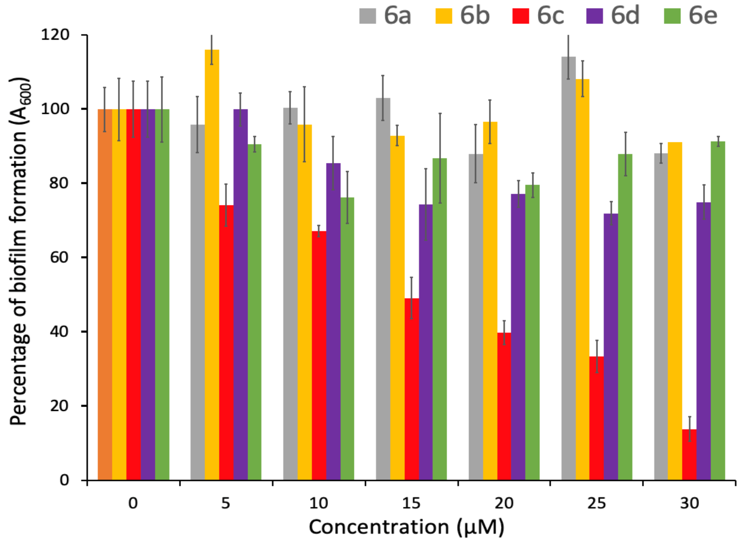
6a 6b 6c 6d 6e
Percentage of biofilm formation (A600)
Concentration (μM)
0 20 40 60 80 100 120
0 5 10 15 20 25 30

c

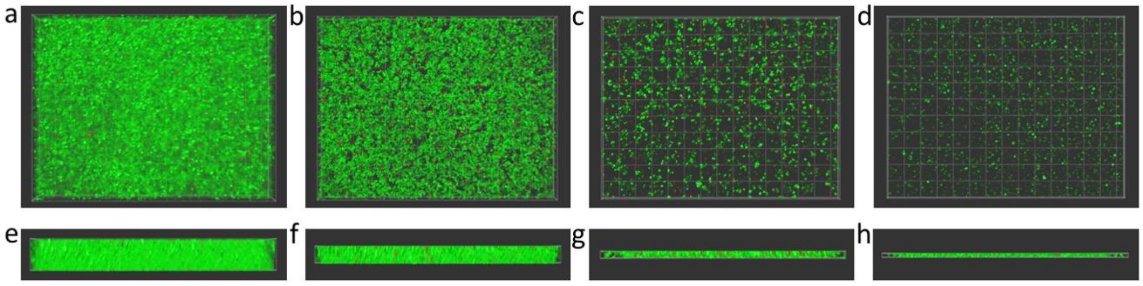
a
b
c
d
e
f
g
h

d

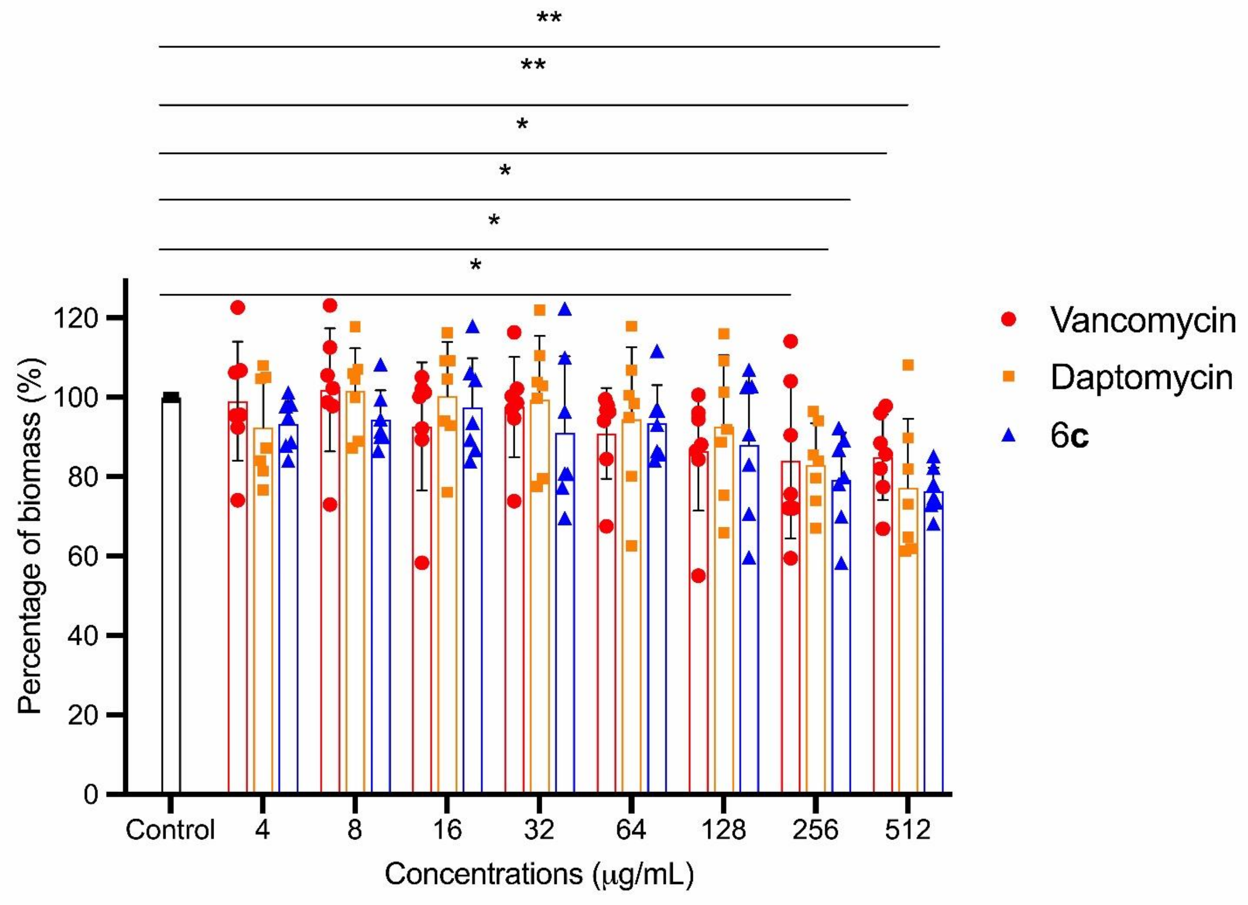

Supplementary Figure 6. Effects of 6c on the inhibition and destruction of biofilm formed by MRSA (ATCC BAA-44). (a-c) Biofilm inhibition assay. (a) Percentage of viable bacteria in the biofilm formed by MRSA in response to the treatment of lawsone-derivatives at varying concentrations (0-30 μM) (mean ± s.d, n=3 independent experiments). (b) The mass of biofilm quantified by crystal violet assay in MRSA in response to the treatment of lawsone-derivatives at varying concentrations (0-30 μM) (mean ± s.d, n=3 independent experiments). **(c)** The fluorescence images of MRSA bacterial cells treated with varying concentrations of compound 6c: (a) media only, (b) 10 μM, (c) 20 μM, (d) 30 μM of compound 6c. The biofilm was stained with the LIVE/DEAD assay kit including SYTO 9 and propidium iodide; e-h represent the side views of each image. (d) Biofilm destruction assay. MRSA biofilm was established on 96-well plate under static culture condition for 24 h and then varying concentrations of 6c, vancomycin, or daptomycin (4-512 μg/mL) were treated to the biofilm for 24 h. The mass of biofilm was then quantified by a crystal violet assay (mean ± s.d, n=7 independent experiments). *: p<0.05, **: p<0.01.

a

Empirical Formula	$C_{168}H_{156}Fe_4O_{28}$
T (K)	180
Space Group	$P\bar{1}$
a (Å)	16.6596(5)
b (Å)	18.9597(5)
c (Å)	24.6232(7)
Cell angles	α = 81.102(2)<br>β = 80.609(2)<br>γ = 72.552(2)
Cell volume (Å^3)	7273.9(4)
Z	2
R-factor (%)	18.2

b

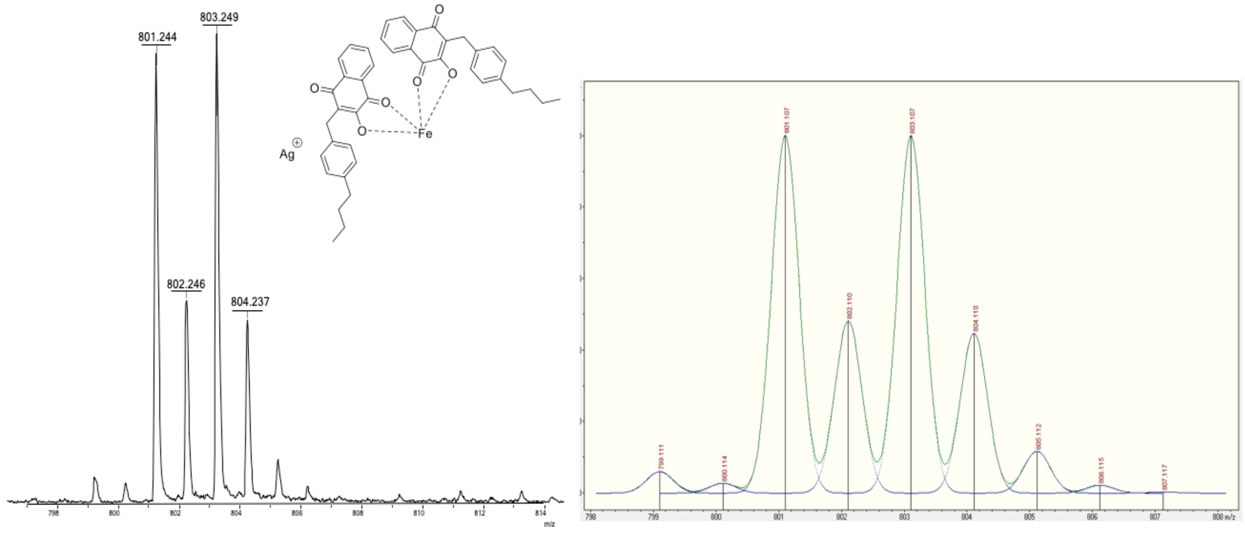

Supplementary Figure 7. **(a)** The crystallographic information of iron-**6c** complex. **(b)** The identity of iron-**6c** complex by MALDI-MS analysis. Left: measured signal in the iron-6c spectrum; Right: simulated spectrum of $[C_{42}H_{38}FeO_6+Ag]^+$. MALDI-MS m/z: $[M+Ag]^+$; Calcd for $[C_{42}H_{38}FeO_6+Ag]^+$ 801.107; found 801.244. The isotopic patterns are consistent between the simulated result and the experimental one, confirming the presence of iron-**6c** complex.

a

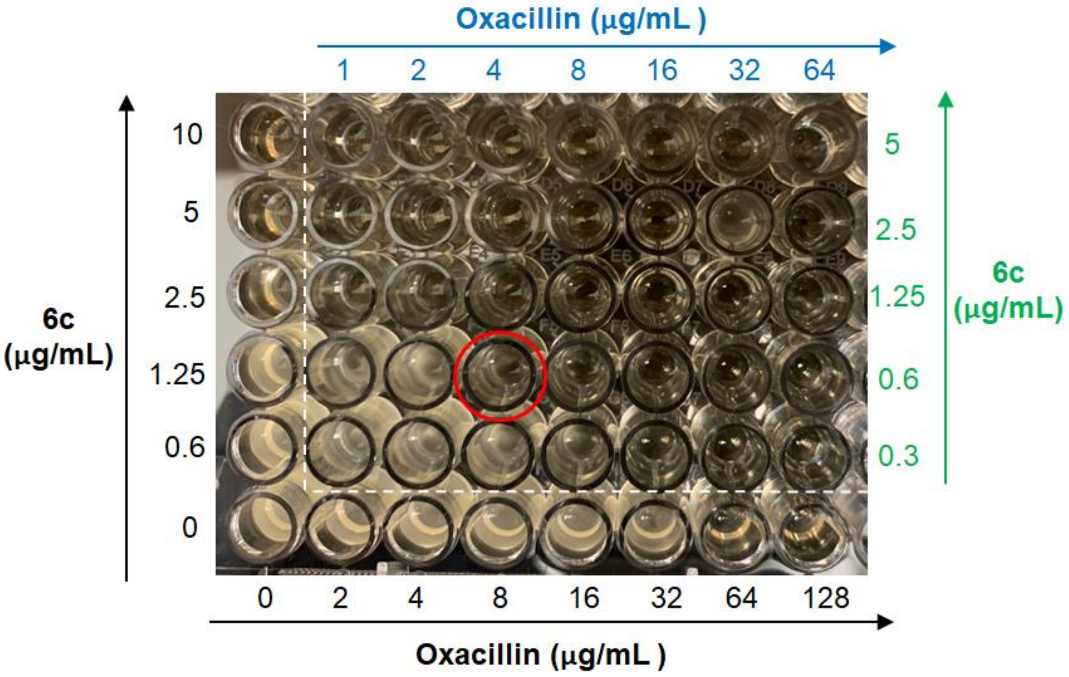

b

MICs against MRSA (ATCC BAA-44)				FIC index
Oxacillin only	Oxacillin with **6c**[a]	**6c** only	**6c** with Oxacillin[b]	
64 μg/mL	4 μg/mL	2.5 μg/mL	0.6 μg/mL	0.31

[a] MIC of oxacillin in the presence of 0.6 μg/mL of **6c**.
[b] MIC of **6c** in the presence of 4 μg/mL of oxacillin.

Supplementary Figure 8. **Checkerboard assay for compound 6c and antibiotic oxacillin against MRSA**. **(**a**)** A representative photograph of MIC assay for combination of **6c** and oxacillin against MRSA (ATCC BAA-44). The black arrow and font in the left column indicate the concentration of **6c** used for the determination of MIC of **6c** only against MRSA, where the MIC was determined to be 2.5 μg/mL. The black arrow and font in the bottom row indicate the concentration of oxacillin used for the determination of MIC of oxacillin only against MRSA, where the MIC was determined to be 64 μg/mL. The green arrow/font and blue arrow/font indicate the concentration of **6c** and oxacillin, respectively, used for the determination of MIC for combination of oxacillin and **6c**, where the MIC was determined to be of 4 mg/mL of oxacillin in the presence of 0.6 mg/mL of **6c** (red circled-well). This assay was repeated twice independently yielding similar results. **(**b**)** The determination of MIC values and FIC index of **6c** and oxacillin against MRSA.

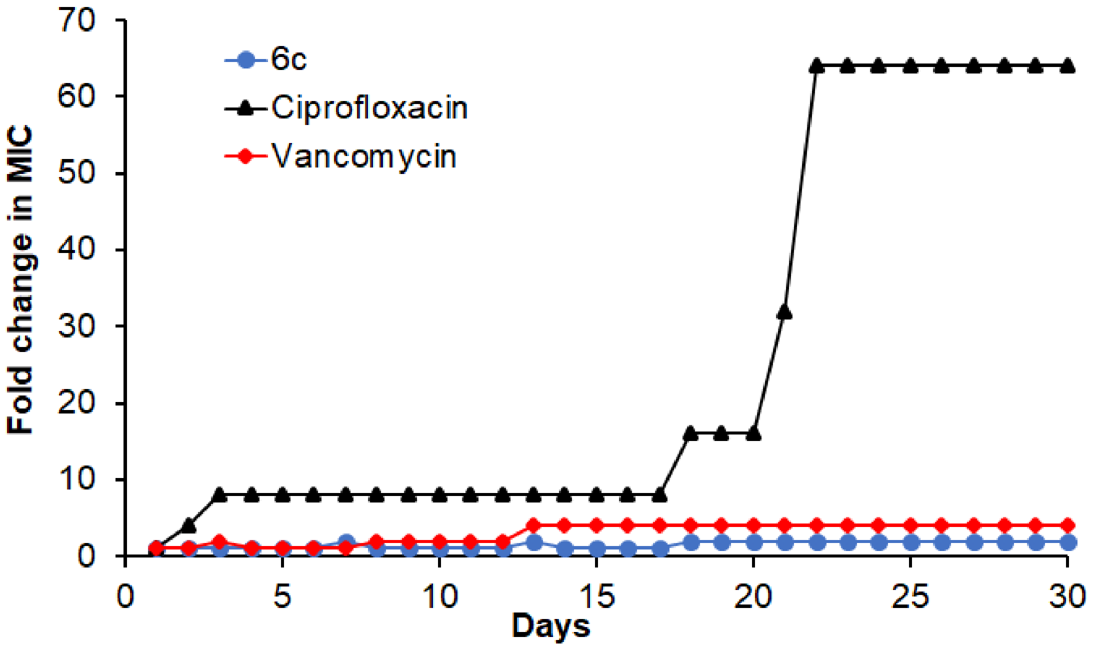

Supplementary Figure 9. In vitro assay for the development of resistance in MSSA towards 6c. The development of resistance in serial passage of MSSA (ATCC 29213 strain) with repeated exposure of ciprofloxacin, vancomycin and **6c** over 30 days. This assay was repeated three times independently yielding similar results.

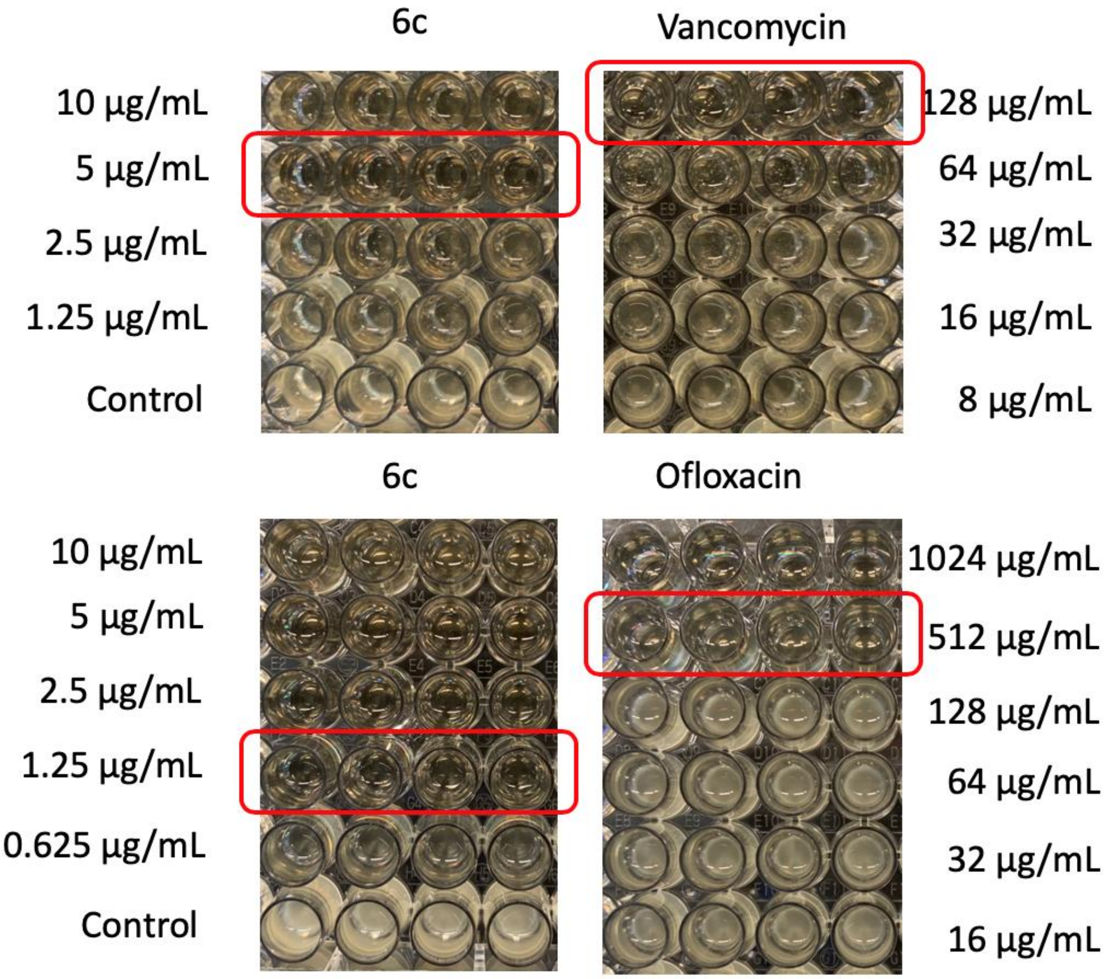

Supplementary Figure 10. Determination of MIC of 6c and antibiotics (Vancomycin or Ofloxacin) against mutant MRSA that had become resistant to ofloxacin (MRSA^{oflR}) or vancomycin (MRSA^{vanR}). Top: Photographs of MIC assay for 6c and vancomycin against MRSA^{vanR} . MICs of 6c and vancomycin were determined to be 5 µg/mL and 128 µg/mL, respectively. Bottom: Photographs of MIC assay for 6c and ofloxacin against MRSA^{oflR} . resistant MRSA (MIC of Vancomycin = 128 µg/mL). MICs of 6c and ofloxacin were determined to be 1.25 µg/mL and 512 µg/mL, respectively.

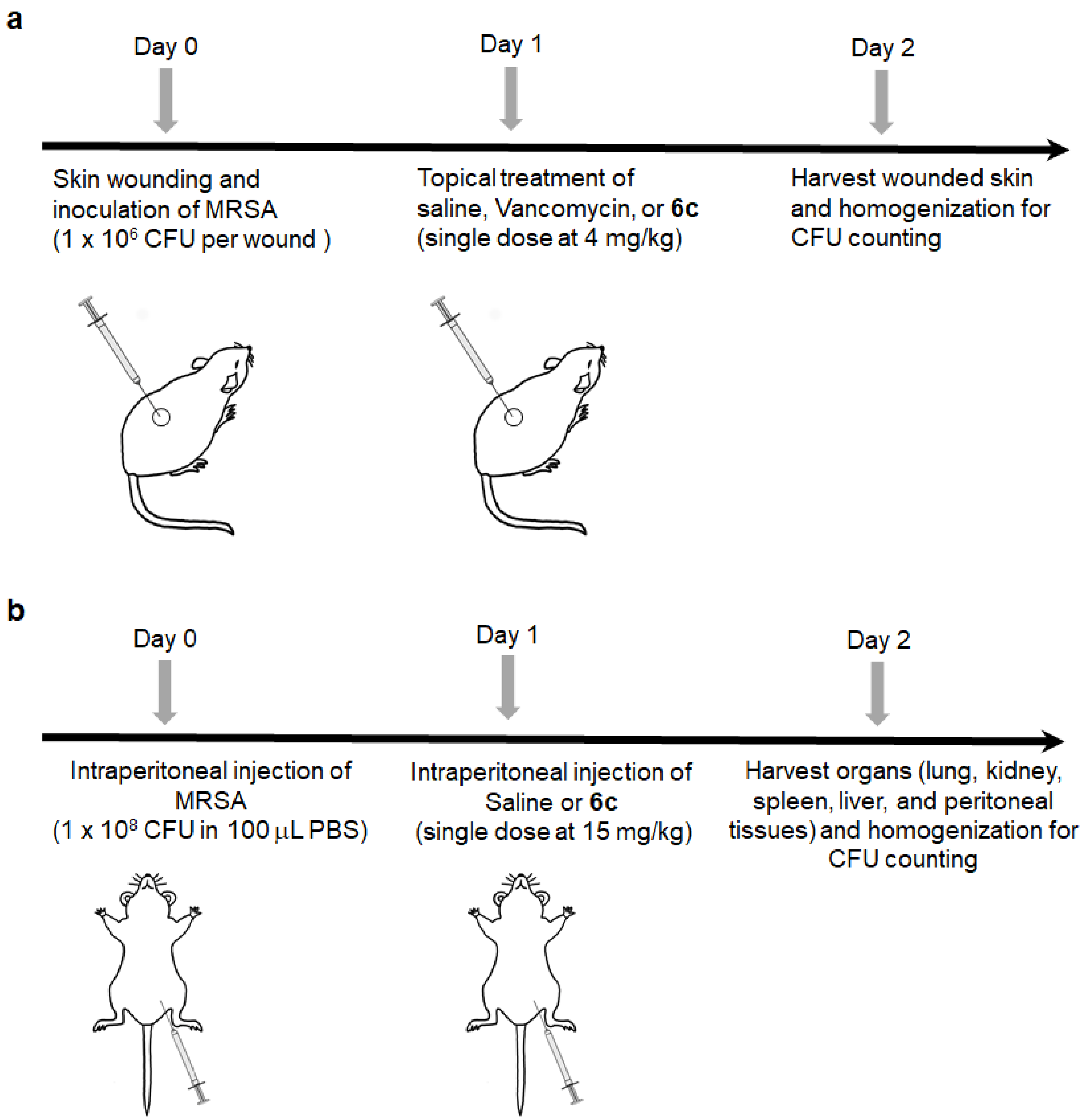

Supplementary Figure 11. Schematics on the experimental procedures for testing in vivo antibacterial efficacy of 6c compound against MRSA in murine models of skin wound infection and peritoneal infection. (a) An experimental procedure for skin wound infection model in mice (C57BL/6 mice). **(b)** An experimental procedure for non-lethal peritoneal infection model in mice (C57BL/6 mice).

Supplementary References

1. STOE und Cie GmbH, X-AREA (2006).
2. Farrugia, L.J. *WinGX* suite for small-molecule single-crystal crystallography. *J Appl Cryst* **32**, 837–838 (1999).
3. Sheldrick, G.M. A short history of *SHELX*. *Acta Cryst* **64**, 112–122 (2008).
4. Macrae, C.F. et al. Mercury CSD 2.0-new features for the visualization and investigation of crystal structures. *J Appl Cryst*., **41**, 466-470 (2008).
5. Lucas, N.T., Notaras, E.G., Cifuentes, M.P., & Humphrey, M.G. Mixed-Metal Cluster Chemistry. 21. Synthesis and Crystallographic and Electrochemical Studies of Alkyne-Coordinated Group 6− Iridium Clusters Linked by Phenylenevinylene Groups. *Organometallics* **22**, 284-301 (2003).
6. Worm-Leonhard, K., & Meldal, M. Green Catalysts: Solid-Phase Peptide Carbene Ligands in Aqueous Transition-Metal Catalysis. *European Journal of Organic Chemistry* **31**, 5244-5253 (2008).
7. Kumar, D., Raj, K. K., Bailey, M., Alling, T., Parish, T., & Rawat, D. S. Antimycobacterial activity evaluation, time-kill kinetic and 3D-QSAR study of C-(3-aminomethyl-cyclohexyl)-methylamine derivatives. *Bioorganic & medicinal chemistry letters* **23**, 1365-1369 (2013).
8. Frahn, J., & Schlüter, A. D. Functionalized AB-type monomers for Suzuki polycondensation. *Synthesis* **11**, 1301-1304 (1997).
9. Ramachary, D. B., Anif Pasha, M., & Thirupathi, G. Organocatalytic Asymmetric Formal [3+2] Cycloaddition as a Versatile Platform to Access Methanobenzo [7] annulenes. *Angewandte Chemie International Edition* **56**, 12930-12934 (2017).
10. Grillo, A. S. et al. Restored iron transport by a small molecule promotes absorption and hemoglobinization in animals. *Science* **356**, 608-616 (2017).
