# Supplementary material for: Naphthoquinone-derivative as a synthetic compound to overcome the antibiotic resistance of methicillin-resistant S. aureus
Source: Commun Biol. 2020 Sep 24;3:529. doi: 10.1038/s42003-020-01261-0 (PMC7518446; doi:10.1038/s42003-020-01261-0)
Supplement: Supplementary file 2 — Description of Additional Supplementary Files [file 42003_2020_1261_MOESM2_ESM.docx]

Description of Additional Supplementary Files

Supplementary Data 1: The atom coordinates and displacement parameters of the iron6c complex.

Supplementary Data 2: Source data for figures.
