# Supplementary material for: Naphthoquinone-derivative as a synthetic compound to overcome the antibiotic resistance of methicillin-resistant S. aureus
Source: Commun Biol. 2020 Sep 24;3:529. doi: 10.1038/s42003-020-01261-0 (PMC7518446; doi:10.1038/s42003-020-01261-0)
Supplement: Supplementary file 5 — Reporting Summary [file 42003_2020_1261_MOESM5_ESM.pdf]

nature research

Corresponding author(s): Min-Ho Kim

Last updated by author(s): Aug 9, 2020

Reporting Summary

Nature Research wishes to improve the reproducibility of the work that we publish. This form provides structure for consistency and transparency in reporting. For further information on Nature Research policies, see our Editorial Policies and the Editorial Policy Checklist.

Statistics

For all statistical analyses, confirm that the following items are present in the figure legend, table legend, main text, or Methods section.

n/a	Confirmed	
☐	☒	The exact sample size (*n*) for each experimental group/condition, given as a discrete number and unit of measurement
☐	☒	A statement on whether measurements were taken from distinct samples or whether the same sample was measured repeatedly
☐	☒	The statistical test(s) used AND whether they are one- or two-sided<br>*Only common tests should be described solely by name; describe more complex techniques in the Methods section.*
☐	☒	A description of all covariates tested
☐	☒	A description of any assumptions or corrections, such as tests of normality and adjustment for multiple comparisons
☐	☒	A full description of the statistical parameters including central tendency (e.g. means) or other basic estimates (e.g. regression coefficient) AND variation (e.g. standard deviation) or associated estimates of uncertainty (e.g. confidence intervals)
☐	☒	For null hypothesis testing, the test statistic (e.g. *F*, *t*, *r*) with confidence intervals, effect sizes, degrees of freedom and *P* value noted<br>*Give P values as exact values whenever suitable.*
☒	☐	For Bayesian analysis, information on the choice of priors and Markov chain Monte Carlo settings
☒	☐	For hierarchical and complex designs, identification of the appropriate level for tests and full reporting of outcomes
☒	☐	Estimates of effect sizes (e.g. Cohen's *d*, Pearson's *r*), indicating how they were calculated

Our web collection on statistics for biologists contains articles on many of the points above.

Software and code

Policy information about availability of computer code

Data collection	The nuclear magnetic resonance spectrums were acquired from a Bruker DMX 400 MHz NMR (Frequency: 400 MHz for lH-NMR; 100 MHz for 13C-NMR); SEM images were obtained from FEI Quanta450 FEG SEM; An inverted Olympus IX83 microscope was used to acquire fluorescence images; Absorbance was measured from SpectraMax multi-mode Spectra Max M4 plate reader (Molecular Devices); An Exactive Plus mass spectrometer (Thermo Scientific, Bremen, Germany) was used to record the high-resolution mass spectra.
Data analysis	Data analysis was performed with Graph Pad Prism 8 (GraphPad) and is stated in the manuscript. The ACD/Percepta software (Ver 2019.1.3, Advanced Chemistry Development, Inc) was used to calculate the ClogP, ClogD7.4 and the number of rotatable bonds; The molecular operating system 2019 was used to calculate globularity.

For manuscripts utilizing custom algorithms or software that are central to the research but not yet described in published literature, software must be made available to editors and reviewers. We strongly encourage code deposition in a community repository (e.g. GitHub). See the Nature Research guidelines for submitting code & software for further information.

Data

Policy information about availability of data

All manuscripts must include a data availability statement. This statement should provide the following information, where applicable:

- Accession codes, unique identifiers, or web links for publicly available datasets
- A list of figures that have associated raw data
- A description of any restrictions on data availability

The XRD crystal data have been deposited in the Cambridge Structural Database (Deposition Number: 1989180, 1989181, 1989182, 1989183, 1989184). All source data underlying the graphs presented in the main figures are available in the Supplementary Data 2. Other data or information that support the findings of this study are available from the corresponding author upon request.

Field-specific reporting

Please select the one below that is the best fit for your research. If you are not sure, read the appropriate sections before making your selection.

☒ Life sciences ☐ Behavioural & social sciences ☐ Ecological, evolutionary & environmental sciences

For a reference copy of the document with all sections, see nature.com/documents/nr-reporting-summary-flat.pdf

Life sciences study design

All studies must disclose on these points even when the disclosure is negative.

Sample size	Sample size was not predetermined using a statistical method, rather it was determined to be adequate based on the magnitude and consistency of measurable differences between groups.
Data exclusions	No data was excluded.
Replication	The in vitro cell culture experiments were typically run in triplicate (3 biological replicates, 3 technical replicates for each biological replicate). For in vivo mice experiments, 3-8 different mice (all C57BL/6 mice from Jackson Laboratory) were used for each experimental group (3 mice per group for skin wound infection protocol and 8 mice per group for peritoneal infection protocol). The CFU counts from homogenized tissue collected from each mouse was run in duplicate and the results were presented as the mean values of the duplicates.
Randomization	Randomization is not relevant to in vitro study involving bacterial cell culture since same bacterial cell is aliquoted and each aliquot received a different treatment with all the appropriate controls running side-by-side. For mice experiments for skin wound infection and peritoneal infection, mice were randomly divided into experimental groups.
Blinding	Blinding was not performed for in vitro bacterial cell culture study, where all measurements are not subject to investigator's bias or ambiguity since they were either machine collected (for example, absorbance or fluorescence) or by directly counting discrete colonies of bacterial cells. In the mice experiments, separate investigators were responsible for conducting treatment protocols and collecting data (bacterial CFU counting from homogenized tissue).

Reporting for specific materials, systems and methods

We require information from authors about some types of materials, experimental systems and methods used in many studies. Here, indicate whether each material, system or method listed is relevant to your study. If you are not sure if a list item applies to your research, read the appropriate section before selecting a response.

Materials & experimental systems

n/a	Involved in the study	
☒	☐	Antibodies
☐	☒	Eukaryotic cell lines
☒	☐	Palaeontology and archaeology
☐	☒	Animals and other organisms
☒	☐	Human research participants
☒	☐	Clinical data
☒	☐	Dual use research of concern

Methods

n/a	Involved in the study	
☒	☐	ChIP-seq
☒	☐	Flow cytometry
☒	☐	MRI-based neuroimaging

Eukaryotic cell lines

Policy information about cell lines

Cell line source(s)	Human fibroblast cell line (ATCC - cat. PCS-201-010) was purchased from American Type Culture Collection and used for mammalian cell viability assay.
Authentication	The cell line was not independently authenticated.
Mycoplasma contamination	The mycoplasma contamination was tested by the supplier.
Commonly misidentified lines (See ICLAC register)	N/A

Animals and other organisms

Policy information about studies involving animals; ARRIVE guidelines recommended for reporting animal research

Laboratory animals	C57BL/6 mice (male mice, 8-12 weeks old) were purchased from Jackson laboratory.
Wild animals	The study did not involve wild animals.
Field-collected samples	The study did not involve samples collected from the field.
Ethics oversight	The experimental protocol was reviewed and approved by the Institutional Animal Care and Use Committee of Kent State University.

Note that full information on the approval of the study protocol must also be provided in the manuscript.
